# Factors and Situations Affecting the Value of Patient Preference Studies: Semi-Structured Interviews in Europe and the US

**DOI:** 10.3389/fphar.2019.01009

**Published:** 2019-09-18

**Authors:** Chiara Whichello, Eline van Overbeeke, Rosanne Janssens, Karin Schölin Bywall, Selena Russo, Jorien Veldwijk, Irina Cleemput, Juhaeri Juhaeri, Bennett Levitan, Jürgen Kübler, Meredith Smith, Richard Hermann, Matthias Englbrecht, Axel J. Hueber, Alina Comanescu, Sarah Harding, Steven Simoens, Isabelle Huys, Esther W. de Bekker-Grob

**Affiliations:** ^1^Erasmus School of Health Policy & Management and Erasmus Choice Modelling Centre, Erasmus University, Rotterdam, Netherlands; ^2^Department of Pharmaceutical and Pharmacological Sciences, University of Leuven, Leuven, Belgium; ^3^Center for Research Ethics & Bioethics, Uppsala University, Uppsala, Sweden; ^4^Applied Research Division for Cognitive and Psychological Science, IEO European Institute of Oncology IRCCS, Milan, Italy; ^5^Belgian Health Care Knowledge Centre, Brussels, Belgium; ^6^Sanofi, Bridgewater, NJ, United States; ^7^Global R&D Epidemiology, Janssen Research & Development, Titusville, United States; ^8^Quantitative Scientific Consulting, Marburg, Germany; ^9^Global Patient Safety and Labeling, Amgen Inc., Thousand Oaks, CA, United States; ^10^Astra Zeneca, Gaithersburg, MD, United States; ^11^Department of Internal Medicine 3 – Rheumatology and Immunology, Friedrich-Alexander-Universität Erlangen-Nürnberg (FAU) and Universitätsklinikum Erlangen, Erlangen, Germany; ^12^Community Health Association, Bucharest, Romania; ^13^Global Patient Safety, Takeda, London, United Kingdom

**Keywords:** patient preferences, drug life cycle, decision-making, health technology assessment, benefit risk assessment, market authorization

## Abstract

**Objectives:** Patient preference information (PPI) is gaining recognition among the pharmaceutical industry, regulatory authorities, and health technology assessment (HTA) bodies/payers for use in assessments and decision-making along the medical product lifecycle (MPLC). This study aimed to identify factors and situations that influence the value of patient preference studies (PPS) in decision-making along the MPLC according to different stakeholders.

**Methods:** Semi-structured interviews (n = 143) were conducted with six different stakeholder groups (physicians, academics, industry representatives, regulators, HTA/payer representatives, and a combined group of patients, caregivers, and patient representatives) from seven European countries (the United Kingdom, Sweden, Italy, Romania, Germany, France, and the Netherlands) and the United States. Framework analysis was performed using NVivo 11 software.

**Results:** Fifteen factors affecting the value of PPS in the MPLC were identified. These are related to: study organization (expertise, financial resources, study duration, ethics and good practices, patient centeredness), study design (examining patient and/or other preferences, ensuring representativeness, matching method to research question, matching method to MPLC stage, validity and reliability, cognitive burden, patient education, attribute development), and study conduct (patients’ ability/willingness to participate and preference heterogeneity). Three types of situations affecting the use of PPS results were identified (stakeholder acceptance, market situations, and clinical situations).

**Conclusion:** The factors and situation types affecting the value of PPS, as identified in this study, need to be considered when designing and conducting PPS in order to promote the integration of PPI into decision-making along the MPLC.

## Introduction

Recently, the use of patient preference information (PPI) in drug development and assessments has gained attention (Meredith et al., 2016). PPI is defined by the United States (US) Food and Drug Administration (FDA) as: “qualitative or quantitative assessments of the relative desirability or acceptability to patients of specified alternatives or choices among outcomes or other attributes that differ among alternative health interventions” (FDA, 2016). Key stakeholders, including the pharmaceutical and medical device industry, regulatory authorities, health technology assessment (HTA)/payers, reimbursement agencies, academia, healthcare professionals, and patient representatives, are recognizing the value of PPI in assessments and decision-making along the medical product lifecycle (MPLC) (Medical Device Innovation Consortium (MDIC), 2015; Abelson et al., 2016).

PPI can be used in every phase of the MPLC (Medical Device Innovation Consortium (MDIC), 2015; Marsh, 2016). Some examples of applications include: identifying unmet medical needs (Medical Device Innovation Consortium (MDIC), 2015; FDA, 2016; Selig, 2016), informing the selection of endpoints, and informing benefit-risk assessments (Medical Device Innovation Consortium (MDIC), 2015; FDA, 2016; Stamuli et al., 2017; Bloom et al., 2018) and in HTA (Kievit et al., 2017; Muhlbacher and Sadler, 2017; Marsh et al., 2018). PPI can give insights into the trade-offs that patients make between benefits and risks and show the relative importance of outcomes for patients (Puhan et al., 2015; Minion et al., 2016; Marsh et al., 2017).

PPI can be obtained through many different preference exploration (qualitative) or elicitation (quantitative) methods (Hockley et al., 2014; Medical Device Innovation Consortium (MDIC), 2015; FDA, 2016; Gutknecht et al., 2016). Exploration methods are recommended for concept exploration and gaining in-depth knowledge on the value of medical products (Facey et al., 2010; Egbrink and IJzerman, 2014; Medical Device Innovation Consortium (MDIC), 2015). Elicitation methods can quantify personal preferences and can allow for statistical analysis and possibly the detection of preference heterogeneity among patients (Puhan MA, 2012; Medical Device Innovation Consortium (MDIC), 2015; FDA, 2016).

Despite prior guidance on the review and research practices of patient preference studies (PPS) (FDA, 2016; Medical Device Innovation Consortium (MDIC), 2015; Bridges et al., 2011), systematic integration and acceptance of PPI into decision-making are still pending. Most industry, HTA/payers and regulators “have key uncertainties regarding the validity, representativeness, and robustness of preference studies to inform deliberative decision-making” (de Bekker-Grob et al., 2017). Therefore, structured insights from all relevant stakeholders on the design and conduct of PPS are currently needed. This study aimed to gather stakeholders’ views on important factors and situations that influence the value and role of PPS in assessments and decision-making. By addressing these factors and situations, decision-makers can be advised on how to conduct and integrate more robust PPS, increasing the potential for their integration along the MPLC.

## Methods

### Design

Interviews were conducted with six stakeholder groups: industry, HTA/payers, regulators, academia, physicians, and a combined group of patients, patient representatives, or patients’ caregivers. Interviews were conducted in eight countries with heterogeneous healthcare systems. Interviews with patients, patient representatives, caregivers, and physicians were conducted in different disease contexts (**Table 1**). Higher interview quota (n = 24) were set for “primary” countries than represented four different regions of Europe. A smaller quota (n = 12) were set for “auxiliary” countries, which served to confirm observations from the primary countries (**Table 1**).

**Table 1:** Planned design of the interviews.

Country classes	Country	Interview quota	Interview quota per stakeholder group*	Disease context**
Primary countries (with a 24 interviewee quota)	Italy	24	4	Lung cancer
	Romania	24	4	Cardiovascular disease
	Sweden	24	4	Rheumatoid arthritis
	United Kingdom	24	4	Muscular dystrophy
Auxiliary countries (with a 12 interviewee quota)	France	12	2	Lung cancer
	Germany	12	2	Rheumatoid arthritis
	Netherlands	12	2	Muscular dystrophy
	United States	12	2	Cardiovascular disease

*Six stakeholder groups were interviewed in every country. These stakeholder groups included the pharmaceutical and medical device industry, HTA/payers, regulators, academia, physicians, and the combined group of patient representatives, patients, and patients’ caregivers.

**Only for interviews with patients, patient representatives, caregivers, and physicians.

### Participant Recruitment

Participants were selected based on predefined inclusion criteria (**Supplemental Material: Appendices I-II**) to enable selection of interviewees with the necessary experience or expertise to answer our research questions and promoting heterogeneity across the sample. Our interviewees were recruited through purposive sampling and snowballing recruitment techniques (Morgan, 2008).

### Conduct

Two interview guides (**Supplemental Material: Appendices IV-V**) were developed based on the research questions and a literature review (van Overbeeke et al., 2019). One guide using technical language was written for industry, regulators, HTA/payer representatives, and academics, and one using plain language for patients, caregivers, physicians, and patient representatives. The interview guides were translated from English into six other languages. Interviews with patient representatives, patients, caregivers, and physicians were conducted in their native language. These guides were translated back to English for a consistency check. Interviews with others were conducted in English, unless they only felt comfortable speaking their native language. To ensure comprehension of the guides, five reviews of the guide and pilot interviews were conducted by targeted stakeholder members. Written informed consent was obtained from all participants before the start of the interviews. The interviews were conducted *via* telephone, teleconference, or face-to-face. Interviews were audio-recorded, transcribed verbatim, and pseudonymized. Non-English transcripts were translated to English.

### Analysis

Transcripts were analyzed through framework analysis (Ritchie and Spencer, 2002; Gale et al., 2013) using NVivo (QSR International Pty Ltd., 2012). The data were interpreted for patterns, consensuses, and critical observations across the stakeholder groups, which created thematic “codes.” First, 2 researchers (CW; EvO) examined 54 transcripts during a “familiarization process” (Ritchie and Spencer, 2002) through which an overview of the collected data was established, and the researchers became aware of key themes and concepts which are made into thematic “codes.” Due to the volume of data, it is common for researchers conducting framework analysis to only familiarize themselves with a section of data (Wolka et al., 2017); in this case, roughly one third of the transcripts (n = 54) was used for familiarization. Next, a thematic framework was identified. Coding was applied to six transcripts (one from each stakeholder group) by two researchers (CW; EvO), in order to confirm the codes identified from the familiarization process, and also to confirm deductive codes formed from a previous systematic review regarding factors and situations influencing the value of PPS (van Overbeeke et al., 2019). Additional open-coding originating from these 6 transcripts were combined with the familiarization process codes and the literature codes to create a final coding list (**Supplemental Material: Appendix VI**), which was then applied to all 143 transcripts by 5 researchers (CW, EvO; KB; RH; MS). Portions or sections of text that corresponded to a theme were indexed and placed in charts with headings reflecting these themes. These charts were then analyzed and interpreted for common attitudes and opinions of the respondents, with comparisons being made inside stakeholder groups and countries.

## Results

**Figure 1:**
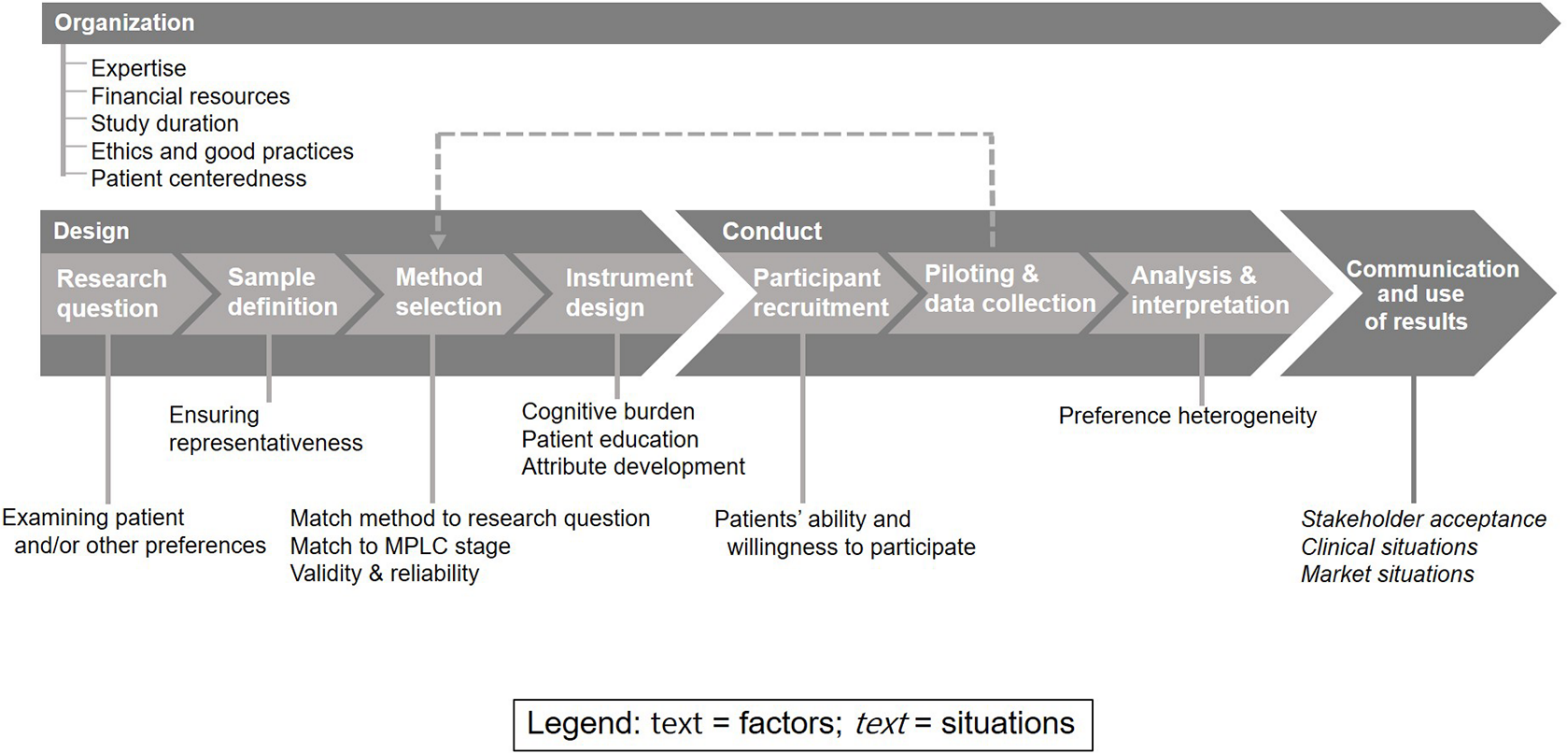
Factors and situations influencing the value and role of patient preference studies (PPS) along the medical product lifecycle (MPLC).

In total, **143 interviews were conducted**, and interview quota was reached for all stakeholder groups and countries except for Romanian regulators (**Table 2**). Factors were identified along three stages of PPS: **organization**, **design**, and **conduct** (**Figure 1**). In addition, challenges and solutions relating to these factors were identified (**Table 3**). Situations were identified during the last stage: **communication and use of results**. These stages were identified through literature (van Overbeeke et al., 2019) and confirmed through interview responses.

**Table 2:** Characteristics of interviewees.

Interviewees (n = 143)	Patients, patient repr. and caregivers (n = 24)		Industry repr. (n = 24)		Regulators (n = 23)		HTA/payer repr. (n = 24)		Physicians (n = 24)		Academics (n = 24)	
**n**	**%**	**n**	**%**	**n**	**%**	**n**	**%**	**n**	**%**	**n**	**%**
*Country*												
Italy (n = 24)	4	17%	4	17%	4	17%	4	17%	4	17%	4	17%
Romania (n = 23)	4	17%	4	17%	3	13%	4	17%	4	17%	4	17%
Sweden (n = 24)	4	17%	4	17%	4	17%	4	17%	4	17%	4	17%
UK (n = 24)	4	17%	4	17%	4	17%	4	17%	4	17%	4	17%
France (n = 12)	2	8%	2	8%	2	9%	2	8%	2	8%	2	8%
Germany (n = 12)	2	8%	2	8%	2	9%	2	8%	2	8%	2	8%
Netherlands (n = 12)	2	8%	2	8%	2	9%	2	8%	2	8%	2	8%
US (n = 12)	2	8%	2	8%	2	9%	2	8%	2	8%	2	8%
Disease area*												
Lung cancer	6	25%	NA		NA		NA		6	25%	NA	
Rheumatoid arthritis	6	25%	NA		NA		NA		6	25%	NA	
Muscular dystrophy	6	25%	NA		NA		NA		6	25%	NA	
Cardiovasc. disease	6	25%	NA		NA		NA		6	25%	NA	
*Self-reported familiarity with PP*												
Very familiar/Expert	1	4%	4	17%	3	13%	2	8%	0	0%	12	50%
Moderately familiar	7	29%	11	46%	13	57%	16	67%	6	25%	7	29%
Not familiar	16	67%	9	38%	7	30%	6	24%	18	75%	5	21%

Cardiovasc., cardiovascular; NA, not applicable; PP, patient preferences; Reg., regulators; repr., representatives; HTA, Health Technology Assessment; UK, United Kingdom; US, United States; *specific disease areas w here only applicable to patients, patient representatives, caregivers, and physicians.

**Table 3:** Challenges and solutions identified in the factors affecting the value of patient preference studies (PPS).

	Factors	Challenge	Solution
**Organization factors**	Expertise	PP studies are complex and need to have correct analysis and interpretation.	Have a multidisciplinary team that has experience with the disease in question and patient preference study conduct and/or methodologies.
	Financial resources	PP studies are costly, and funding is difficult to obtain.	Ensure enough budget to not compromise recruitment and sample size.
	Study duration	Patient recruitment is time consuming, particularly with rare diseases.	Consider recruiting through many multiple channels (healthcare professionals, internet calls, patient organizations, national registries, and clinical trials). It is possible to conduct a complete patient preference study between 6 and 12 months.
	Patient centeredness	Patient centeredness needs to be integrated into study design.	Allow patients and/or patient representatives to be involved in the design of PP studies.
	Ethics and good practices	Obtaining ethical approval is often a barrier for researchers. Privacy concerns might make patients hesitant to participate. Stakeholders are concerned about industry bias.	Ensure ethical approval is always obtained. Ensure anonymous, confidential data collection and be transparent about how patient data is handled. Involve a neutral party to balance potential biases.
**Design factors**	Examining patient and/or other preferences	It is not always known whose preferences need to be studied.	This largely depends on the research question; if patient preferences need to be known, then the sample should consist out of patients. In some cases, however, PP can be supplemented with caregiver/public preferences
	Ensuring representativeness	Ensuring representativeness and generalizability is difficult when preferences are largely subjective.	Where possible, examine a large sample size and include patients that have experience with or sufficient knowledge about the disease and are informed about treatments and/or drug development.
	Matching method to research question	Selecting a method appropriate for a research question	There is no “gold standard” method, and matching a method to the research question depends on what is attempting to be measured, and what form the results need to be. Some favor methods that can handle hypothetical scenarios, can quantify trade-offs, or include monetary valuations.
	Matching method to MPLC stage	Selecting a method appropriate for an MPLC stage	Qualitative methods are thought to be more appropriate for earlier stages, to identify attributes, and then followed up with quantitative methods to quantify these attributes at later stages.
	Validity and reliability	Validity is important and needs to be maintained.	Scientific consensus on an international level needs to standardize validity, including method, criteria, internal, and external validity. Despite there being no “gold standard” method, there still needs to be a standardized way to evaluate all methods.
	Cognitive burden	Cognitive burden needs to be taken into consideration to involve patients with all ranges of abilities.	Try to limit the use of elements that add greater cognitive burden: hypothetical scenarios, complex probabilities, or percentages, describing complex stages of the MPLC, repeating the same type of question many times, long surveys/interviews with no breaks, abstract ideas without concrete examples, and surveys that cannot be completed at home.
	Patient education	Patients’ knowledge is often not sufficient to contribute meaningful answers.	Utilize educational tools, perhaps in video or other format, to instruct patient preference exercises.
	Attribute development	Attributes need to contain important information for decision-makers, but also be comprehensible to patients.	Too many attributes in one choice task in some elicitation methods, such as a DCE, can become burdensome to patients. Ensure easily interpretable attributes by involving patients and/or representatives in their construction.
**Conduct factors**	Patient ability and willingness to participate	Participant recruitment is difficult if patients are unable or unwilling to participate.	Recruit patients through multiple channels (healthcare professionals, internet calls, patient organizations, national registries, and clinical trials) and offer studies that patients can complete at home, or offer travel reimbursement schemes.
	Preference heterogeneity	Heterogeneity in PP studies is difficult to address in decision-making.	Heterogeneity is inherent and must be sufficiently captured or quantified through PP exploration or elicitation or methods.

### Factors Relating to Study Organization

Five factors relating to the **organization** of PPS were identified, namely: (1) expertise of staff, (2) financial resources, (3) study duration, (4) ethics and good practices, and (5) patient centeredness (**Figure 1**, **Table 3**). The first four factors were anticipated and will not be exhaustively detailed in the results, because they are factors that influence the value of any scientific study. Although they are important factors, they are not unique to PPS. Further information about these factors’ current implementation in PPS is summarized in the systematic review by van Overbeeke et al. (van Overbeeke et al., 2019). The fifth factor, patient centeredness, was more specific to PPS. Patient centeredness in study organization is key to a successful PPS, since this should be the focus of the study. Patients are invaluable to informing the research questions, improving recruitment, and ensuring the comprehensibility of information and questions given to patients (van Overbeeke et al., 2019). Interviewees across all stakeholder groups and countries, except for Romania, expressed the importance of having patients and/or patient representatives involved in the design and conduct, because they have the most real-life experience with their own condition.

### Factors Relating to Study Design

Eight factors relating to the **design** of PPS were identified (**Figure 1**). All stakeholder groups across all countries felt that study design is a crucial phase of PPS.

1. Examining patient and/or other preferences
  All interviewees were asked whose preferences should be measured, and their answers included patients, patient organizations, the public, caregivers, physicians, and combinations of these categories. Four interviewees discussed how the population must satisfy the research question. The preferences of family caregivers could be important in instances when caregivers make most of the medical decisions because patients cannot express themselves or their preferences may not be reliable (e.g., children or impaired cognitive abilities). Although caregiver preferences can sometimes reflect patient preferences, they also have their own preferences which could be different from the patient’s preferences. HTA/payer representatives, patients, industry representatives, and academics discussed preferring general public preferences instead of PPI, or a combination thereof, in the context of healthcare expenditure, budget allocation, and reimbursement. A UK HTA/payer representative stated they must consider “*all of the uses of the NHS [National Health Service] rather than just the particular patient group who is in front of them*.” Five HTA/payer representatives assumed that patient preferences are incorporated satisfactorily and “*built in*” (UK) through QALYs or EQ5D measures, despite their calculation frequently incorporating public utility values for quality of life attributes as opposed to patient utilities for treatment outcomes. An EU industry representative stated that when it is the objective to elicit patient preferences, the general public should not be included.
2. Ensuring representativeness
  Representatives from industry, HTA/payers, regulatory, academia, patients, and patient organizations across all countries expressed that it is crucial to have a patient sample representative of the actual patient population targeted with the treatment. All stakeholder groups across all countries had opinions about what constitutes as ideal characteristics of the patient sample, although this depends largely on the research question. Almost all participants agreed that patients should have experience with the disease, although this is not as important in contexts like disease prevention. Eighteen participants believed participants should have experience with treatment, stating that this results in more “*reliable*” (UK, regulator) preferences. Fourteen participants stated experience with treatment was less important. Other characteristics of an ideal sample included covering multiple countries of residence, if generalizability across countries is desired. Six interviewees (industry, regulatory, academia, and HTA/payer) expressed concern that the subjective nature of PPI generates a lower generalizability. US and EU academics, an EU HTA/payer representative, and an EU regulator explained how recruitment through patient organizations can lead to samples of highly motivated patients, and recruitment through clinical trials can give samples restricted by predetermined inclusion or exclusion criteria, both of which are not representative of the patient population. A solution, according to the regulator, might be to recruit 50% patient organizations and 50% “*everyday*” patients.
3. Matching the method to the research question
  The majority of industry, HTA/payer, regulators, and academic representatives mentioned that there is no single “*gold standard*” preference exploration (qualitative) or elicitation (quantitative) method. According to one academic, selecting a method is in itself “*a trade-off*” because “*none of them are perfect … [or] offer you a guarantee of being better over any other one”* (UK). The ability to quantify trade-offs was thought to be paramount in elicitation methods by many stakeholder groups, particularly academics. An EU physician was adamant that an elicitation method needs to be equipped to handle hypothetical scenarios, including drugs that are not yet available, and must include the option to not receive any treatment. This physician felt this was extremely applicable to conditions that have limited treatment options. One EU academic required elicitation methods to assess marginal rate of substitution to determine maximum acceptable risk. Five academics and one HTA/payer representative from Sweden and Germany advocated for elicitation methods that incorporate a monetary valuation, such as willingness-to-pay. EU academic and industry representatives indicated that elicitation methods are seen as more “*robust”* (US, industry), “*objective”* (Germany, industry), and “*acceptable by [sic] the medical … community”* (Netherlands, academic) and more likely to “*convince a payer*” (France, academic).
4. Matching the method to the MPLC stage
  Exploration methods were thought better suited for early MPLC phases for exploring heterogeneous data, defining value frameworks, or identifying endpoints or attributes, examining cultural heterogeneity and subjective experiences. Most interviewees from academia and industry argued that a mixed-method approach is necessary, discovering attributes and levels early in the MPLC through exploration methods to “*inform*” (US, academia) elicitation studies during phase III or IV. Elicitation methods were thought to “*refine quality [ … ] with numbers”* (Italy, regulator) by quantifying trade-offs, obtaining the strength of a preference, ranking or weighing an endpoint, and/or comparing decision pathways. Two UK HTA/payer representatives mentioned that they are most familiar with elicitation methods during appraisals.
5. Validity and reliability
  Most interviewees from all countries, especially HTA/payer representatives and academics, felt validity was extremely important, including method validity, criteria validity, internal validity, and external validity. EU HTA/payer representatives, a regulator, and two industry representatives expressed a need for international scientific consensus regarding evaluating rigor of methodologies in a “*standardized, validated way—accepted by everybody, all stakeholders*” (France). One academic assured that studies published in articles undergo quality checklists for validity but also suggested that piloting should be conducted within studies for quality and validity checks. A US regulator pointed out that some methods have higher validity than others and suggested that the degree of rigor should be “*fit for purpose*,” meaning the level of validity should be proportional to the risk or invasiveness of the medical product. A US industry member explained how external validity is often not necessary for internal decision-making. An EU academic is worried that use of hypothetical situations leads to low external validity.
6. Cognitive burden
  All stakeholder groups emphasized that cognitive abilities of patients must be considered when designing preference studies, particularly for diseases associated with cognitive impairment or neuromuscular fatigue. This assures validity of the data but also places value in the preferences of all patients, regardless of cognitive ability. Many interviewees insisted that patients with cognitive issues should not be excluded, with an EU physician stating that stakeholders “*have to get [sic] extra input to try and find out exactly what their views are*” (UK). A physician, regulatory, and HTA/payer representative mentioned that survey fatigue should be avoided. Two patients said that patients often have different preferences depending on how they are feeling that particular day (UK patient and UK caregiver). Several cognitively burdensome situations were highlighted by academics, regulators, industry, and HTA representatives, including (1) hypothetical scenarios, (2) complex probabilities or percentages, (3) complex stages of the drug life cycle, (4) many repetitions of similar questions with different phrasing, (5) long surveys with no breaks, (6) absence of concrete examples, and (7) surveys that cannot be completed at home. A US academic expressed, *“a poorly designed study that was poorly understood by the users could result in a worse decision than if there had been no study what-so-ever.”*
7. Patient education
  Four EU and US industry members commented that patients’ knowledge is often not sufficient to contribute meaningful answers. However, other stakeholders, including patients themselves, recognized that the responsibility of patient education does not lie with the patients, but with the study designers, who should ensure comprehensibility. An EU industry member advised utilizing educational tools, in video or other format, to instruct patient preference exercises. A US academic stated “*we need to figure out how to make the instrument as comprehensible as possible to respondents who may have a very low educational attainment but still be accurate enough that we can map the attributes and levels to clinical outcomes*,” expressing the need to strike a right balance. Two EU stakeholders recognized that informing patients without contributing bias or “*influenc[ing]”* the patient (Germany, physician) is “*a challenge”* (Sweden, regulator).
8. Attribute development
  All stakeholders who expressed an ideal number of attributes for PPI elicitation studies thought that it should be between three and six attributes. Any more than seven, and the cognitive burden can become unmanageable, or the patients “*[simplify] characteristics*” (US, industry), affecting validity. Both EU and US interviewees mentioned standard attributes to incorporate like quality of life, overall survival rates, progression-free survival rates, or other symptomatic evaluations. An EU academic also advocated for the inclusion of “willingness-to-pay” (WTP) features, as well as the level of convenience of managing conditions. An EU physician suggested to include the products’ administration frequency. Most representatives from different stakeholder groups emphasized the importance of constructing “*easily interpretable*” (Netherlands, regulator) and “*patient-user friendly*” (UK, regulator) questions, thereby measuring their preferences accurately. Two EU representatives spoke about making sure not to “*steer certain answers*” (UK, industry) and recognized that patients can be susceptible to the particular question ordering. A US academic strongly warned against using ambiguous attribute levels such as “*mild, moderate, and severe*.” A US regulator explained *“Patients who had experience of the disease should be essential in determining the type of questions, and for example, attributes [ … ] also, the wordings and the communication part”*.

### Factors Relating to Study Conduct

Two factors relating to the **conduct** of PPS will be examined (**Figure 1**), namely: (1) patients’ ability and willingness to participate and (2) preference heterogeneity.

1. Patients’ ability and willingness to participate
  Interviewees identified different hurdles to patient recruitment including lack of resources, the possibility of people dropping out (or passing away) during the course of recruitment, fraudulent participants, and a hesitation of patients to discuss traumatic elements of their disease or condition. EU patients, a caregiver, and an industry representative said newly diagnosed patients or patients in severe, late stages of illness might not want to participate. In the context of myotonic dystrophy, an EU caregiver explained *“It can take a long time for them to accept [the diagnosis] and to understand it”* (UK). A US HTA/payer representative stated *“One of the biggest issues is finding the patient”* and explained that, normally, 1 month is given for recruitment, although two EU academics and two EU physicians stated that it can take years for rare diseases. Multiple channels for recruitment were proposed by interviewees including healthcare professionals, internet calls, patient organizations, national registries, and clinical trials. To facilitate recruitment, two EU patients have suggested to conduct studies from home or reimburse travel and hotel costs.
2. Preference heterogeneity
  Almost all interviewees argued that preference heterogeneity, or the extend that preferences vary across individuals, is inevitable and “*inherent*” (UK, industry) due to differences in treatment experience, disease experience, cultures and countries, education, socio-economic status, family and support systems, and beliefs and religions. Two regulators (EU), two academics (EU and US), and three industry representatives (EU) stated that capturing heterogeneity is complex and is often difficult to integrate into decision-making. Four industry representatives (EU and US), three regulators (EU), and three HTA/payers (EU) argued that heterogeneity is actually desirable and is “*a gift”* (HTA/payer, Germany). It enables subgroup identification and exploration of opinions. An EU regulator stated that heterogeneity is only a problem if *not* taken into account. Three EU regulators and a US industry representative stated that heterogeneity investigation requires larger sample sizes. A patient, academic, and a regulator (EU and US) argued that PPI should be measured at multiple points in time to examine preference variation. An EU academic stated that preference studies should always conduct a latent class or other model of standard deviation to explore preference heterogeneity and allow for subgroup analysis.

### Situations Relating to Communication and Use of PPS Results

Three types of **situations** were identified that may have an influence on the use of PPS results in decision-making along the MPLC (**Figure 1**), including (1) stakeholder acceptance, (2) clinical situations, and (3) market situations. Participants were asked directly whether there are any situations where the use of patient preferences could be more useful or less useful, and the following situations were discussed.

1. Stakeholder acceptance
  Stakeholder acceptance, or the lack thereof, is a significant situation that can affect the use of PPS results along the MPLC. A patient representative, HTA/payer representative, and academics (EU, US) expressed that a *“cultural change”* (UK, HTA/payer) is needed to have assessors look at these data, because they are often *“narrow-minded”* (US, academia) about considering PPI. A patient and two regulators expressed the need for training assessors on the topic of PPI. Several interviewees, particularly HTA/payers and regulators, were reluctant towards PPI and gave more importance to other criteria, such as cost-effectiveness and clinical outcomes. Three interviewees questioned the ability of patients to provide valuable input since they are *“not rational,” “too emotional,”* (Romania, academic) and *“not able to make the best decisions for themselves”* (US, HTA/payer). Almost half of Romanian interviewees mentioned that PPI do not have a place in the MPLC and was “*never [ … ] important in Romania*” (industry).
2. Clinical situations
  PPI was perceived to be particularly useful in the context of chronic diseases, rare diseases, remission disorders, high unmet medical needs, unclear benefit-risk balance (both in terms of moderate benefits/high risks or heterogeneous drug profiles), limited availability of clinical (or other) information, end-of-life treatments, invasive treatments, and treatments with subjective endpoints. Two EU regulators believed that PPI is less important in the case of medical devices, especially the less invasive they are. An EU patient stated the opposite, explaining that patients have to use these devices every day. Two EU regulators and an EU academic believed that new or innovative products would benefit from PPI, although two other EU regulators disagreed, stating that patients would not be familiar enough with these products. Other contexts where PPI was perceived to be less useful include biological fluids, diagnostics, non-invasive treatments and medical devices, prevention medicine, surgical interventions, therapies with no clinical evidence (such as homeopathy), clear differences between new product and reference, new drugs with no added value compared to those on the market, acute disorders (excluding cancer), or situations with a clear benefit-risk balance.
3. Market situations
  Two EU industry representatives and one US HTA/payer representative stated that PPI would be especially important in crowded therapeutic markets where trade-offs will be made between products, although one EU HTA/payer representative believed PPI would not add significant value in this situation. An EU industry representative stated that PPI would be less useful during significant changes in the therapeutic market. An EU regulator stated that they would also not be as important when no medical alternatives are available, like for with orphan drugs, although patient acceptance to the treatment can still be measured. Another EU regulator stated that it might not be useful to examine PPI on sensitive topics with public controversy.

## Discussion

Through 143 semi-structured interviews with 6 stakeholder groups in 8 countries, we identified 15 factors and 3 types of situations that can influence the value and role of patient preferences in assessments and decision-making along the MPLC. Our results offer key insights into the barriers hindering the integration of patient preferences throughout assessments and decision-making in the MPLC, and our interviewees also expressed solutions as to how these challenges can be overcome (**Table 3**). Frequently cited factors included matching method to research question and MPLC stage, validity, cognitive burden, and preference heterogeneity.

Our results confirm the statements of the FDA guidance on PPI (FDA, 2016) and other studies (Postmus et al., 2016; Wolka et al., 2017) regarding the involvement of patients and patient representatives in the design and conduct of preference studies. This leads to a better comprehensibility of the questions given to patients, improves recruitment, and ensures correct interpretation and communication of results. The FDA guidance on PPI (FDA, 2016) also indicates that exploration methods would be most useful during discovery, which is confirmed by our results. Our interviewees believed elicitation methods were suited for later phases in the MPLC although some argued that mixed-method studies would be beneficial. The use of elicitation methods is well documented in regulatory benefit-risk assessments (van Til and Ijzerman, 2014) and HTAs (Weernink et al., 2014). However, HTA/payers also often utilize qualitative PPI or patient involvement (Mott, 2018), which was also supported by our results.

Interviewees provided insightful guidance into the various elements of a patient preference study (PPS) that could increase cognitive burden, such as hypothetical scenarios and complex probabilities or percentages, which the FDA guidance on PPI also includes (FDA, 2016). Some interviewees preferred at-home surveys, although several authors (Tervonen et al., 2017; Postmus et al., 2018) argue that survey administration *via* interviews or workshops can provide beneficial in-person support to patients. Some interviewees highlighted the importance of always using patient preferences instead of public utilities, except in situations where proxies to patients may be required. Others emphasized using public utilities in economic evaluations to ensure the applicability to more general allocation decisions. The issue of public *versus* patient preferences in health economic evaluations remains a pressing issue of debate (Faggioli et al., 2011; FDA, 2016).

Interviewees indicated that there is a lack of guidance on the validity assessment of preference methods, confirming similar findings in the MDIC PCBR report (Medical Device Innovation Consortium (MDIC), 2015). Conceptual models created for validity assessments of some methods are currently being developed (Janssen et al., 2017). Capturing preference heterogeneity and allowing for the identification for subpopulations will increase the value of PPS for benefit-risk assessments and HTA (Medical Device Innovation Consortium (MDIC), 2015; FDA, 2016; Postmus et al., 2016; Kievit et al., 2017). Interviewees suggested that capturing preference heterogeneity requires larger sample sizes and the use of latent class or other models of standard deviation. Janssen et al. (2017) also concluded that advanced statistical methods might improve the understanding of preference heterogeneity in benefit-risk assessments, although the number of subpopulations that can be evaluated is limited.

The identified market situations influencing the value of PPI support the concept of preference sensitive decisions, described by the MDIC PCBR report as “[decisions] … in which there are multiple diagnostic or treatment options, and the decision which option to pursue depends upon the particular preferences of the decision maker” (Medical Device Innovation Consortium (MDIC), 2015). They encompass situations when no option is clearly superior over a plausible range of preferences and/or the evidence supporting one option over others is considerably uncertain. In addition, our findings confirm the statements of Hollin *et al*. (Hollin et al., 2017), the FDA guidance on PPI (FDA, 2016), and the MDIC PCBR report (Medical Device Innovation Consortium (MDIC), 2015) that populations with unmet medical needs and rare diseases are specific situations where PPI is very valuable.

### Strengths and Limitations

This study’s fundamental strength originates from interviewing international experts in a variety of fields relating to every stage of the MPLC. Additionally, we interviewed patients, caregivers, and patient representatives, representing potential participants in PPS.

Recruitment of interviewees was mostly done through the PREFER consortium and snowballing. Participants might have had an upfront interest PPI, creating a non-random sampling bias. A limited sample of each stakeholder group per country was interviewed, thus is not representative of the whole stakeholder population. The self-reported familiarity with the topic of PPI differed among stakeholder groups and countries (**Table 2**). Participants with low self-reported familiarity may have provided less informative insights. In Romania, 74% of interviewees stated they were “not familiar” with PPI. However, all participants were recruited based on their significant experience as members of key stakeholder groups in the MPLC. Even if a participant had limited, direct experience with PPS, their experience with different stages of the MPLC, the incorporation of evidential data at these stages, or their interaction with patients, is highly relevant when considering the potential for PPI integration, and the factors that might be supporting or hindering this integration.

The semi-structured nature of the interviews created a flexible rapport between interviewer and interviewee, allowing follow-up questions to be asked. However, not all factors and situations were discussed with every interviewee since some opted not to answer questions. For example, due to time constraints, participants were unable to elaborate on the reasons why PPI is useful in some clinical and market situations, and instead listed them.

There was variation between interviews since eight interviewers conducted the interviews in seven different languages. Qualitative methodologies rely on subjective experiences and perspectives of participants in order to observe phenomena with context and detail. Therefore, the results of these interviews represent the thoughts and opinions of the interviewees and may not always be objective or generalizable to every organization or country. However, the large number of 143 interviews contributed to triangulating the data and examining an accurate account of the current status of PPI in the MPLC. Further research is recommended to contextualize these stakeholder comments with each country’s unique healthcare system and identify when PPI can be best integrated into the MPLC.

## Conclusions

Many situations and factors need to be taken into account when designing and conducting PPS in order to obtain valuable results that can be used in decision-making. By examining the guidance given by 143 stakeholders with unique expertise, more robust PPS can be performed in order to incorporate PPI successfully into the MPLC. Moreover, these results can help medical product developers to decide, whether or not and how to include PPS in their development plan, avoiding waste of resources and the unnecessary exhaustion of patient populations.

## Data Availability

The datasets generated for this study are available on request to the corresponding author.

## Ethics Statement

The studies involving human participants were reviewed and approved by Belgium: Medical Ethics Committee of University Hospital (UZ) KU Leuven/Research (S59790), France: Commission Nationale de l’Informatique et des Libertés (CNIL) (2036344), Germany: Ethik-Kommission der Friedrich-Alexander Universität (92_17 B), Italy: Comitato Etico Instituto Europeo di Oncologia (IEO) (R587/17-IEO 609), The Netherlands: Medisch Ethische Toetsings Commissie Erasmus Medical Centre (WT/ss/METC306661), Romania: Comisia de Bioetica a Medicamentului si a Dispozitivelor Medicale (CNBMDM) (5 SNI), Sweden: Regionala Etikprövningsnämnden Uppsala (EPN) (2017/001/1), United Kingdom: Newcastle University Ethics Committee (11307/2016), United States: Western Institutional Review Board (WIRB) (1-1010535-1). The patients/participants provided their written informed consent to participate in this study.

## Author Contributions

All authors contributed to the protocol design of this study and/or the acquisition of the data. CW, EO, RJ, SR, KS, AC, and ME conducted the interviews. CW and EO performed the analysis and wrote the initial draft. EB-G, IH and JV were involved in the further refinement of the main text and figures. All authors reviewed the study materials and findings. All authors read and approved the final version before submission.

## Funding

The Patient Preferences in Benefit-Risk Assessments during the Drug Life Cycle (PREFER) project has received funding from the Innovative Medicines Initiative 2 Joint Undertaking under grant agreement No 115966. This Joint Undertaking receives support from the European Union’s Horizon 2020 research and innovation programme and EFPIA.

## Conflict of Interest Statement

The Patient Preferences in Benefit-Risk Assessments during the Drug Life Cycle (PREFER) project has received funding from the Innovative Medicines Initiative 2 Joint Undertaking under grant agreement No 115966. This Joint Undertaking receives support from the European Union’s Horizon 2020 research and innovation programme and EFPIA. This text and its contents reflect the PREFER project’s view and not the view of IMI, the European Union or EFPIA. JJ declares the following competing interests: employee of Sanofi, a global biopharmaceutical company focused on human health; and ownership of shares in Sanofi and in investment portfolio which at times includes other pharmaceutical and healthcare-related companies. BL declares the following competing interests: employee of Janssen Research and Development, LLC; and stockholder in Johnson & Johnson and in a portfolio that at times includes other pharmaceutical and healthcare-related companies. JK declares the following competing interests: representing Quantitative Scientific Consulting, Marburg, Germany; Scientific Consultant working for the pharmaceutical industry; and stockholder in a portfolio that includes pharmaceutical and healthcare-related companies. SH is an employee of Takeda. MS is an employee of Amgen. RH is an employee of AstraZeneca.

The remaining authors declare that the research was conducted in the absence of any commercial or financial relationships that could be construed as a potential conflict of interest.
