# Supplementary material for: Factors and Situations Affecting the Value of Patient Preference Studies: Semi-Structured Interviews in Europe and the US
Source: Front Pharmacol. 2019 Sep 18;10:1009. doi: 10.3389/fphar.2019.01009 (PMC6759933; doi:10.3389/fphar.2019.01009)
Supplement: Supplementary file 1 [file Table_1.docx]

**Appendix I**

**Recruitment Protocol: Sweden, Romania, Italy, UK**

|  | **Regulatory Authorities*** | **HTA/payers*^1^** | **Patient, caregivers and patient organisation*** | **Physicians*^2^** | **Academics*** | **Industry*^3^** |
| --- | --- | --- | --- | --- | --- | --- |
| **Sweden** | N=2 Representatives of Swedish Medical Products Agency with i) a formal role in healthcare products regulation process AND ii) experience in regulatory affairs as well as knowledge of national regulations N=2 Swedish representatives with a role or official position within EMA OR ii) collaborations and/or interactions with European regulatory agencies for the Evaluation of Medicinal Products AND ii) knowledge of European legislation | N=4 Representatives of i) a Swedish Health Technology Assessment Organization or a Swedish reimbursement agency; OR ii) who are involved in the evaluation of a health technology OR in the prescription drugs and health care reimbursement decision-making procedures. | Target disease: Rheumatoid Arthritis N=1 Swedish representative of one of the main (Swedish) Rheumatoid Arthritis patient organisations N=1 Patient over 18 newly diagnosed with RA (within 6 months) N=1 Patient over 18 on at least one synthetic DMARD N=1 Patient over 18 on at least one biological DMARD | Target disease: Rheumatoid Arthritis N=4 Rheumatologists OR primary care provider OR physicians working with Rheumatoid Arthritis patients OR physicians as part of a medical team caring for Rheumatoid Arthritis patients in Sweden | N=4 persons working in a Sweden academic/research institution with knowledge/experience in the drug life cycle OR patient preference methods | n=2/3 persons whose activities are focused on pharmaceuticals  n=1/2 persons whose activities are focused on medical devices |
| **Romania** | N=2 Representatives of Romanian National Agency for Medicines and Medical Devices with i) a formal role in healthcare products regulation process AND ii) experience in regulatory affairs as well as knowledge of national regulations  N=2 Romanian representatives with a role or official position within EMA OR ii) collaborations and/or interactions with European regulatory agencies for the Evaluation of Medicinal Products AND ii) knowledge of European legislation | N=4 Representatives of i) a Romanian Health Technology Assessment Organization or a reimbursement agency; OR ii) who are involved in the evaluation of a health technology OR in the prescription drugs and health care reimbursement decision-making procedures. | Target disease: Cardiovascular Diseases  N=1 Romanian representative of one of the main (Romanian) Cardiovascular diseases patient organisations N=1 Patient i) aged ≥ 18; ii) recent (<6months) cardiovascular event  N=1 Patient i) aged ≥ 18; ii) diagnosed with a cardiovascular disease; iii) more than 2 years post-event N=1 Patient i) aged ≥ 18; ii) diagnosed with a cardiovascular disease; iii) more than 5 years post-event | Target disease: Cardiovascular Diseases  N=4 Cardiologists OR Cardiothoracic surgeons OR Primary care providers OR physicians working with CVD patients OR physicians as part of a medical team caring for CVD patients in Romania | N=4 persons working in a Romanian academic/research institution with knowledge/experience in the drug life cycle OR patient preference methods | n=2/3 persons whose activities are focused on pharmaceuticals  n=1/2 persons whose activities are focused on medical devices |
| **Italy** | N=2 Representatives of Italian Medicines Agency (AIFA) with i) a formal role in healthcare products regulation process AND ii) experience in regulatory affairs as well as knowledge of national regulations N=2 Italian representatives with a role or official position within EMA OR ii) collaborations and/or interactions with European regulatory agencies for the Evaluation of Medicinal Products AND ii) knowledge of European legislation | N=4 Representatives of i) an Italian Health Technology Assessment Organization or an Italian reimbursement agency; OR ii) who are involved in the evaluation of a health technology OR in the prescription drugs and health care reimbursement decision-making procedures. | Target disease: lung cancer N=1 Italian representative of one of the main (Italian) cancer patient organisations N=1 Patient i) aged ≥ 18; ii) diagnosed with lung cancer within 3 months; and iii) in treatment N=1 Patient i) aged ≥ 18; ii) diagnosed with lung cancer within 1 year; and iii) in treatment N=1 Patient i) aged ≥ 18; ii) diagnosed with lung cancer iii) more than 5 years post-diagnosis; and iv) out of treatment | Target disease: lung cancer N=4 Oncologists OR Surgeon OR Primary care providers OR physicians OR working with lung cancer patients OR physicians as part of a medical team caring for lung cancer patients in Italy | N=4 persons working in an Italian academic/research institution with knowledge/experience in the drug life cycle OR patient preference methods | n=2/3 persons whose activities are focused on pharmaceuticals  n=1/2 persons whose activities are focused on medical devices |
| **UK** | N=2 Representatives of Medicines & Healthcare Products Regulatory Agency with i) a formal role in healthcare products regulation process AND ii) experience in regulatory affairs as well as knowledge of national regulations N=2 UK representatives with a role or official position within EMA OR ii) collaborations and/or interactions with European regulatory agencies for the Evaluation of Medicinal Products AND ii) knowledge of European legislation | N=4 Representatives of i) an UK Health Technology Assessment Organization or a reimbursement agency OR ii) who are involved in the evaluation of a health technology OR in the prescription drugs and health care reimbursement decision-making procedures | Target disease: muscular dystrophy N=1 UK representative of one of the main (UK) muscular dystrophy patient organisations N=1 Patient newly diagnosed (<6months) with muscular dystrophy; i) aged ≥ 18 OR ii) caregiver if patient is younger than 18 or cognitive impaired  N=2 Patient diagnosed with muscular dystrophy; i) aged ≥ 18 OR ii) caregiver if patient is younger than 18 or cognitive impaired | Target disease: muscular dystrophy N=4 Specialists (cardiologists; neurologists; orthopaedists; physiatrists; pulmonologists) OR primary care provider working with Neuromuscular Dystrophy patients OR physicians as part of medical team caring for Neuromuscular Dystrophy patients in the UK | N=4 persons working in an English academic/research institution with knowledge/experience in the drug life cycle OR patient preference methods | n=2/3 persons whose activities are focused on pharmaceuticals  n=1/2 persons whose activities are focused on medical devices |

* Ensure a mix of males and females whenever possible

^1^ Ensure the inclusion of bodies with differing scope and budget responsibilities

^2^ Ensure the inclusion of both physicians of university and local hospitals

^3^ Includes consultants for industry/representatives of general associations of pharmaceutical industry. Ensure the inclusion of both smaller and larger companies

**Appendix II**

**Recruitment Protocol: Netherlands, Germany, France, US**

|  | **Regulatory Authorities*** | **HTA/payers*^1^** | **Patient, caregivers and patient organisation*** | **Physicians*^2^** | **Academics*** | **Industry*^3^** |
| --- | --- | --- | --- | --- | --- | --- |
| **Netherlands** | N=1 Representative of Medicines Evaluation Board (CBG) with i) a formal role in healthcare products regulation process AND ii) experience in regulatory affairs as well as knowledge of national regulations  N=1 Dutch Representative with a role or official position within EMA OR ii) collaborations and/or interactions with European regulatory agencies for the Evaluation of Medicinal Products AND ii) knowledge of European legislation | N=2 Representatives of i) a Dutch Health Technology Assessment Organization or a reimbursement agency; OR ii) who are involved in the evaluation of a health technology OR in the prescription drugs and health care reimbursement decision-making procedures. | Target disease: muscular dystrophy N=1 Dutch representative of one of the main (Dutch) muscular dystrophy patient organisations N=1 Patient diagnosed with muscular dystrophy; i) aged ≥ 18 OR ii) caregiver if patient is younger than 18 or cognitive impaired | Target disease: muscular dystrophy N=2 Specialists (cardiologists; neurologists; orthopaedists; physiatrists; pulmonologists) OR primary care provider working with Neuromuscular Dystrophy patients OR physicians as part of medical team caring for Neuromuscular Dystrophy patients in the Netherlands | N=2 persons working in a Dutch academic/research institution with knowledge/experience in the drug life cycle OR patient preference methods | n=1/2 persons whose activities are focused on pharmaceuticals  n=0/1 persons whose activities are focused on medical devices |
| **Germany** | N=1 Representative of Federal Institute for Drugs and Medical Devices (BfArM) or Paul-Ehrlich-Institut (PEI) - with i) a formal role in healthcare products regulation process AND ii) experience in regulatory affairs as well as knowledge of national regulations  N=1 German representative with a role or official position within EMA OR ii) collaborations and/or interactions with European regulatory agencies for the Evaluation of Medicinal Products AND ii) knowledge of European legislation | N=2 Representatives of i) a German Health Technology Assessment Organization or a reimbursement agency; OR ii) who are involved in the evaluation of a health technology OR in the prescription drugs and health care reimbursement decision-making procedures. | Target disease: Rheumatoid Arthritis N=1 German representative of one of the main (German) Rheumatoid Arthritis patient advocacy organisations N=1 Patient over 18 on at least one biological DMARD | Target disease: Rheumatoid Arthritis N=2 Rheumatologists OR primary care provider OR physicians working with Rheumatoid Arthritis patients OR physicians part of a medical team caring for Rheumatoid Arthritis patients in Germany | N=2 persons working in a German academic/research institution with knowledge/experience in the drug life cycle OR patient preference methods | n=1/2 persons whose activities are focused on pharmaceuticals  n=0/1 persons whose activities are focused on medical devices |
| **France** | N=1 Representative of French National Agency of Medicine and Health Products Safety (ANSM) with i) a formal role in healthcare products regulation process AND ii) experience in regulatory affairs as well as knowledge of national regulations  N=1 French representative with a role or official position within EMA OR ii) collaborations and/or interactions with European regulatory agencies for the Evaluation of Medicinal Products AND ii) knowledge of European legislation | N=2 Representatives of i) a French Health Technology Assessment Organization or a reimbursement agency; OR ii) who are involved in the evaluation of a health technology OR in the prescription drugs and health care reimbursement decision-making procedures. | Target disease: lung cancer N=1 French representative of one of the main (French) cancer patient organisations N=1 Patient i) aged ≥ 18; ii) diagnosed with lung cancer iii) more than 5 years post-diagnosis; and iv) out of treatment | Target disease: lung cancer N=2 Oncologists OR surgeon OR Primary care providers OR physicians OR working with lung cancer patients OR physicians as part of a medical team caring for lung cancer patients in France | N=2 persons working in a French academic/research institution with knowledge/experience in the drug life cycle OR patient preference methods | n=1/2 persons whose activities are focused on pharmaceuticals  n=0/1 persons whose activities are focused on medical devices |
| **US** | N=1 Representative of US Food and Drug Administration (FDA) FDA from the Center for Drug Evaluation and Research (CDER) or the Center for Biologics Evaluation and Research (CBER)  N=1 Representative of US Food and Drug Administration (FDA) from the Center for Devices and Radiological Health (CDRH). | N=2 Representatives of i) an American Health Technology Assessment Organization or a reimbursement agency; OR ii) who are involved in the evaluation of a health technology OR in the prescription drugs and health care reimbursement decision-making procedures. | Target disease: Cardiovascular diseases N=1 US representative of one of the main Cardiovascular diseases patient organisations (in the USA) N=1 Patient i) aged ≥ 18; ii) diagnosed with a cardiovascular disease; iii) more than 2 years post-event | Target disease: Cardiovascular diseases N=2 Cardiologists OR Cardiothoracic surgeons OR Primary care providers OR physicians working with CVD patients OR physicians as part of a medical team caring for CVD patients in the US | N=2 persons working in a North American academic/research institution with knowledge/experience in the drug life cycle OR patient preference methods | n=1/2 persons whose activities are focused on pharmaceuticals  n=0/1 persons whose activities are focused on medical devices |

* Ensure a mix of males and females whenever possible

^1^ Ensure the inclusion of bodies with differing scope and budget responsibilities

^2^ Ensure the inclusion of both physicians of university and local hospitals

^3^ Includes consultants for industry/representatives of general associations of pharmaceutical industry. Ensure the inclusion of both smaller and larger companies

**Appendix III**

**Ethical approval**

This study was approved by nine ethics committees in the eight countries where interviews and focus groups were conducted plus Belgium. The ethics committees (with registration numbers of the approvals):

Belgium: Medical Ethics Committee of University Hospital (UZ) KU Leuven/Research (S59790)

France: Commission Nationale de l'Informatique et des Libertés (CNIL) (2036344)

Germany: Ethik-Kommission der Friedrich-Alexander Universität (92_17 B)

Italy: Comitato Etico Instituto Europeo di Oncologia (IEO) (R587/17-IEO 609)

The Netherlands: Medisch Ethische Toetsings Commissie Erasmus Medical Centre (WT/ss/METC306661)

Romania: Comisia de Bioetica a Medicamentului si a Dispozitivelor Medicale (CNBMDM) (5 SNI)

Sweden: Regionala Etikprövningsnämnden Uppsala (EPN) (2017/001/1)

United Kingdom: Newcastle University Ethics Committee (11307/2016)

United States: Western Institutional Review Board (WIRB) (1-1010535-1)

**Supplemental Material IV**

**Interview guide and glossary for patients, caregivers, patient representatives and physicians**

BLUE = instructions to the interviewer (not to be spoken out loud)

**BOLD = eye-catcher to interviewer (important terms)**

*ITALICS = definition/quote*

# Introduction:

1. Before interview: read briefing on how to conduct interviews on Project Place
2. Present yourself: say your name and that you are a researcher of the IMI PREFER project
3. Thank for participation in advance
4. Briefly explain the aim of the IMI PREFER project and interviews:
   - Project: to **strengthen patient-centric decision-making throughout the life cycle** of drugs and medical devices by **developing recommendations**.
   - Interviews: to **explore the opinions** of different stakeholders about measuring and using patient preferences when making drugs and medical devices.
5. Put interviewee at ease:

- No wrong answers
- Digitally recorded
- Confidential, anonymous
- Interview will be approximately 45 minutes to 1 hour
- Voluntary, do not have to answer anything they do not feel comfortable answering
- They can stop the interview at any time, without having to give a reason

1. Give the informed consent form, if not signed yet
2. Do not give glossary yet! (Glossary can be given after question 2.)
3. Put on audio recorder

Interview Questions:

- 1. Can you tell me **a little bit** more about yourself and your background?
- For patient representatives ONLY: What is your **current role** in the patient organization?

A. Definition of patient preferences

- 1. The PREFER project wants to determine **how** the preferences of patients can be measured when making drugs or medical devices. What does the term ‘patient preferences’ mean to you?

For this interview, we are using the following definition of patient preferences:

*Patient preferences reflect why patients choose a particular health treatment over other available options. This health treatment can be a drug or a medical device. A preference can be stated for a health treatment as a whole or for the advantages and disadvantages of one treatment. In order to make a choice or state a preference, patients need to weigh up the advantages and disadvantages and compare them to those of other health treatments.*

Give glossary to interviewee: There are some terms that we will use here today that you might not use in everyday language, so if there’s anything that you have not heard before, or aren’t too sure about, we have a glossary for you. You can stop and read it at any time.

B. Current status of patient preferences

We have some questions about your thoughts on the current situation of measuring and using patient preferences.

- 1. How **familiar** are you with patient preference studies?
- If patient/caregiver: Have you already taken part in a study on patients’ preferences?
- If physician/patient representative: Do you have experience with designing a patient preference study?

Examples to give: A patient preference study asks patients about what they want from a flu vaccine (flu shot) by asking what matters most to them, such as how effective it is, how long it lasts, its side-effects, where patients can get it, or how much it costs. Some patients might say that the effectiveness of the vaccine is most important, while others think that a vaccine should have the lowest level of side-effects.

- 1. Drugs are made in different stages: first it is made in a laboratory, then the drug is tested in animals and then in humans (these are the clinical trials). Finally, based on the results from these studies, the government decides if the pharmaceutical company can sell the drug. Medical devices are approved in a different way, but have to respect similar requirements.

To your knowledge, **when** are patient preferences currently being measured? At what stage of developing a drug and bringing it to patients?

- Do you know **who** is measuring preferences?
- Do you know what **methods** are being used to measure preferences?

If interviewee get stuck: Methods mean how researchers ask patients for their preferences and then analyse the answers, such as by interview, questionnaire, or asking patients to pick between a series of choices…

- 1. If interviewee has experience with patient preference methods or studies: What were the study results used for? (e.g. in the development of drugs, marketing authorization decisions, reimbursement decisions if applicable, decisions about the price of a product)
  2. If interviewee has experience with patient preference methods or studies: Which challenges did you encounter in:
- In the organization and management of patient preference studies?
- In the design of patient preference studies?
- During the conduct of patient preference studies?
- In the use of the results of patient preference studies?
  1. If interviewee has experience with patient preference methods or studies: Did these challenges influence the usefulness of the patient preference study?
  2. If interviewee has experience with patient preference methods or studies: Did the heterogeneity of the patient sample influence the usefulness of your patient preference study?
- How did you deal with this?
  1. If interviewee has experience with patient preference methods or studies: Under what conditions, situations or circumstances would you think that patient preferences are less important and why?
- Are there circumstances where using patient preferences is counterproductive? Why?

Role of patient preferences: REQUIREMENTS

- 1. In order to set up a patient preference study, first, it has to be decided who will participate in patient preference studies.
- **Who** do you think should participate in patient preference studies?
- **How long** should they have been **experiencing the disease**?
- To what extent should they have **experience with treatment**?
- To what extent do you think that caregivers **reflect** the patient’s preferences?
  1. What else do you think is needed in a patient preference study in order to measure patient preferences?

Role of patient preferences: CONCERNS

- 1. Do you have any concerns about **how** patient preferences are **measured**? `
  2. Do you have any concerns about **companies using** information from patient preference studies?

ex. This information could include a patient’s personal preferences or medical history

- 1. Do you have any concerns about **using** patient preferences at other decision points, (ask if they remember taking about decision points) such as helping the government decide **whether to put the product on the market**, deciding the **price** of a product, (if applicable) and whether it can be **reimbursed**?

D. Round up questions

These were all the questions I had for you, but before we finish:

- Do you have anything you want to **add** or **emphasise**?
- Do you have any **questions for me**?
- Do you have a suggestion for **another interesting interviewee**?
- Would you feel comfortable being contacted again if we have any **follow up questions**?

Thank you for your participation. If you have any other questions, comments, or want to get in touch with me, I will give you my contact details.

Glossary:

List of definitions

- **Patient preferences**

Patient preferences reflect why patients choose a particular health treatment over other available options. This health treatment can be a drug or a medical device. A preference can be stated for a health treatment as a whole or for the advantages and disadvantages of one treatment. In order to make a choice or state a preference, patients need to weigh up the advantages and disadvantages and compare them to those of other health treatments.

- **Clinical trial**

A research with humans to evaluate the efficacy and safety of a medicinal product, device or any other health-related intervention.

- **Decision points**
  The moment during the drug lifecycle at which a decision needs to be made that influences the course of the medical product or device development, reimbursement, or authorization process.

E.g. the decision made by companies to develop the product, decisions made by regulatory agencies whether to put the product on the market, decisions about the price of a product, and whether it can be reimbursed.

- **IMI and the PREFER project**

Project that is supported by the Innovative Medicines Initiative (IMI). IMI is a European initiative that wants to speed the development of better and safer medicines. The PREFER project wants to find out how patient preferences can support decisions when making a medicine or medical device and bringing it to the patient.

- **Medical device**

Any instrument or machine that helps in the treatment or diagnosis of a disease (for example: a pacemaker, a hospital bed, a wheelchair, an asthma inhaler, etc.)

- **Methods (research)**

The way in which researchers set-up and conduct their research. For example, how researchers ask patients for their preferences. This could be done by interview, questionnaire, or surveys where they ask patients to pick between a series of choices.

- **Stakeholders**

Partner organisations or individuals involved in this research that represent:

Patients, academics, Health Technology Assessment bodies^1^, healthcare professionals^2^, patient organisations^3^, pharmaceutical companies^4^, reimbursement agencies, and regulatory authorities^5^.

^1^**Health Technology Assessment body (HTA body)**

Organisation that evaluate the effects of health technologies and impact on society (includes medical products, devices, techniques, procedures and organisational systems in health care).

^2^**Healthcare professional**

A healthcare professional or healthcare provider is an individual who provides healthcare services to people. Healthcare professionals include physicians, nurses, dentists, pharmacists, and a wide variety of other human resources trained to provide some type of healthcare service.

^3^**Patient organization**

Group that consists of people that represent and work with/for patients.

^4^**Pharmaceutical company**

A company that develops, makes, and sells drugs and medical devices.

^5^**Reimbursement agency**

Agencies that decide how a drug or medical device will be financed, either by a national government, a health insurance company, or paid privately by patients. This largely varies from country to country.

⁶**Regulatory authority**

Organisation that **regulate** the market authorisation and approval of new drugs, and provide guidance and support in their research and development.

e.g. EMA in Europe, FDA in the United States

**For example: Industry decision about which product to develop**

**For example: Regulatory marketing authorization decision**

Pre-discovery

Upstream research

Drug Discovery

Preclinical

Clinical drug development

(Phase I, II, III)

Post approval

(Phase IV)

Laboratory and animal testing to investigate if drug is safe

The drug is tested in humans:

**Phase I:** to determine safety and safe dosage range of the drug in a small number of healthy volunteers

**Phase II:** to determine the efficacy of the drug in patients

**Phase III:** confirm previous results and identify side effects in a large number of patients

Post-marketing studies are conducted to assess the safety and effectiveness of the drug on long-term and in the real world

Research for a new drug starts in the laboratory

Experiments are conducted to discover a new drug

**The Drug Lifecycle with examples of decisions**

**For example: Reimbursement decision (if applicable)**

**Supplemental Material V**

**Interview guide for industry representatives, regulatory representatives, HTA/payer representatives, and academics**

BLUE = instructions to the interviewer (not to be spoken out loud)

**BOLD = eye-catcher to interviewer (important terms)**

*ITALICS = definition/quote*

Introduction:

1. Before interview: read briefing on how to conduct interviews on Project Place
2. Present yourself: say your name and that you are a researcher of the IMI PREFER project
3. Thank for participation in advance
4. Briefly explain the aim of the IMI PREFER project and interviews:
   - Project: to **strengthen patient-centric decision-making throughout the life cycle** of drugs and medical devices by **developing recommendations** to guide industry, Regulatory Authorities, and HTA bodies on how and when patient preference studies should be performed and the results can be used to inform decision-making
   - Interviews: to **explore the opinions** of different stakeholders about the assessment of patient preferences and use of patient preference information in the development and evaluation of drugs and medical devices. These stakeholders are patients, patient organization representatives, physicians, industry representatives, academics, regulators, HTA body representatives and reimbursement agency representatives
5. Put interviewee at ease:

- No wrong answers
- Digitally recorded
- Confidential, anonymous
- Interview will be approximately 45 minutes to 1 hour
- Voluntary, do not have to answer anything they do not feel comfortable answering
- They can stop the interview at any time, without having to give a reason

1. Give the informed consent form, if not signed yet
2. Put on audio recorder

Interview Questions:

- 1. Can you tell me **a little bit** more about yourself and your background?
- What is your **current role** in your organization?

A. Definition of patient preferences

- 1. The IMI PREFER project wants to determine **how** patient preferences can be measured for the development and evaluation of drugs and medical devices. How would you **define** the term ‘patient preferences’?

For this interview, we are using the definition of patient preferences used in the FDA guidance for medical devices:

*“The relative desirability or acceptability to patients of specified alternatives or choices among outcomes or other attributes that differ among alternative health interventions”*

In other words, patient preferences are the basis of how patients choose a particular treatment over others. To make a choice, patients make trade-offs between a treatment’s characteristics, weighing its advantages and disadvantages collectively.

Give glossary to interviewee: There are some terms that we will use here today that you might not use in everyday language, so if there’s anything that you have not heard before, or aren’t too sure about, we have a glossary for you. You can stop and read it at any time.

B. Current status of patient preferences

First, we have some questions about your thoughts on the **current situation** of measuring and using patient preferences.

- 1. How **familiar** are you with patient preference studies?
- Do you have experience with designing or conducting patient preference studies?
  1. If interviewee has experience with patient preference methods or studies: Which challenges did you encounter:
- In the organization and management of patient preference studies?
- In the design of patient preference studies?
- During the conduct of patient preference studies?
- In the use of the results?
  1. If interviewee has experience with patient preference methods or studies: Do you consider these challenges to be factors that influence the utility of patient preference studies?
  2. If interviewee has experience with patient preference methods or studies: Was heterogeneity of the patient sample a factor that influenced the utility of your patient preference study?
- How did you deal with this?
  1. In this project, we focus on the drug and medical device life cycle**, starting from discovery to post-marketing surveillance**. Patient preference studies can be conducted in different stages.
- To your knowledge, **at what point** in the drug and medical device life cycle are patient preference studies **currently** being **conducted**?
- Do you know **who** is conducting patient preference studies?
- Do you know what **methods** they are using?
  1. Several **decisions** are made in the drug or medical device life cycle, such as industry decisions about product development, regulatory decisions about marketing authorization and decisions about pricing and reimbursement. To what extent do you think patient preferences are **currently** used for these decisions?
- Which decisions in particular and why?
  1. What are the **current procedures or protocols** to integrate patient preferences in these decisions?
- In the evaluation of drugs and medical devices, how are patient preferences currently compared or combined with other clinical or non-clinical data?
  1. Under what conditions, situations or circumstances are patient preferences **less important** and why?
- Are there circumstances where using patient preferences is **counterproductive**? Why?
- Under what conditions, situations or circumstances are patient preferences **extremely important** and why?
  1. Do you know of any other methods for measuring patient preferences?

Role of patient preferences: REQUIREMENTS

- 1. According to you, **when or at which stage in** the drug and medical device life cycle do you think patient preference studies **should** be **conducted** for informing the decisions we have talked about**?**
  2. We talked about the different decisions in the drug or medical device life cycle where patient preferences **should be** taken into account. According to you, **what is needed** to implement the use of patient preferences at those decisions?
     - - What would help **your organization** to take patient preferences into account more **systematically for decision-making**?
- What is **lacking** now?
  - Do you think a registry for patient preference studies is needed?
  1. Do you think specific protocols or procedures are needed to **integrate** patient preferences into decision-making?
- If so, what protocols are needed for **industry** decision-making?
- If so, what protocols are needed for **marketing authorization** decision-making?
- If so, what protocols are needed for **HTA**?
- If so, what protocols are needed for **reimbursement** decision-making
- If so, what protocols are needed for (...) other decision-making?
  1. In order to use the results from a patient preference study for informing a specific decision, it has to be decided when to start the patient preference study. **How much time should be allocated before a decision point** in order to conduct the patient preference study and to incorporate the results from this study in the decision?
  2. When patient preference studies are designed, it has to be decided will participate in patient preference studies. **Who** do you think should participate in patient preference studies?
- To what extent should they have **experience with the disease**?
- To what extent should they have **experience with treatment**?
- What is your opinion of using caregivers as proxies for patients in patient preference studies?
  - To what extent do you think that they **reflect** the patient’s preferences?
  1. We talked about some patient preference methods. What are your thoughts about the **usefulness** of each method that we have discussed?
- Under what circumstances would you recommend using one **rather than other**?
- Under what circumstances would you recommend using a **quantitative or a qualitative** approach?
  1. To what extent does the **method** need to have high **validity** for you to want to **use** or **consider it for evaluation**? If interviewee is unsure/asks about validity: “*Validity means the extent to which a*[*measurement*](http://www.biology-online.org/dictionary/Measurement)*,*[*test*](http://www.biology-online.org/dictionary/Test)*or*[*study*](http://www.biology-online.org/dictionary/Study) [*measures*](http://www.biology-online.org/dictionary/Measures)*what claims to measure*. *Internal validity refers to whether a finding that incorporates a causal relationship between two or more variables is sound. External validity refers to whether the results of a study can be generalised beyond the specific research context in which the study was conducted. Face validity is a concern with whether an indicator appears to reflect the content of the concept in question.”*
  2. What do you consider to be important indicators for **evaluating the quality of patient preference studies**?
     - How do these **differ** from quality indicators of **evidence from clinical trials**?

Role of patient preferences: CONCERNS

- 1. Do you have any concerns about **how** patient preferences **are measured**?
  2. Do you have any concerns about **using** patient preferences in decision-making?
- Do you have any concerns about using patient preferences specifically **by your organization**?
  1. Is **heterogeneity** a concern when **conducting or assessing** patient preference studies?
- If so, how important?

D. Round-up questions

These were all the questions I had for you, but before we finish:

- Do you have anything you want to **add** or **emphasise**?
- Do you have any **questions for me**?
- Do you have a suggestion for **another interesting interviewee**?
- Would you feel comfortable being contacted again if we have any **follow-up questions**?

Thank you for your participation. If you have any other questions, comments, or want to get in touch with me, I will give you my contact details.

**Appendix VI**

**Coding list applied to transcripts**

| Organization |
| --- |
| Expertise - Staff |
| Financial resources |
| Study duration |
| Patient preference study design |
| Ethics - Good science rules compliance |
| Objective - RQ - Perspective (individual PP vs group PP vs caregiver PP) |
| Patient centeredness in PP studies |
| Patient population (including age, sex, type, duration of illness) |
| Heterogeneity |
| Participant recruitment process |
| Representativeness - Generalisability |
| Preference exploration elicitation instrument design |
| Attributes |
| Cognitive Complexity - cognitive ability - learning tools or instruments |
| Number of Attributes |
| Question framing |
| Preference exploration- elicitation method selection |
| Outcome Measurements (including risk tolerance, uncertainty, stated preferences, tradeoffs) |
| Validity - Reproducibility |
| Use of the results from patient preference studies |
| Not useful |
| Useful |
| Processes currently being used |
| HTA & reimbursement (including Economic evaluation, Cost-effectiveness analysis, Cost-benefit analysis, Cost-utility analysis, PRO identification, Outcomes weighing, QALY estimation) |
| Industry |
| Clinical development (including Clinical trial design, Patient Relevant Outcomes (PRO) identification, Treatment arm selection, Product design validation, Patient trade-off understanding) |
| Pre-clinical development (including Product design validation) |
| Research and discovery (including Ideation, Unmet medical need identification, Prototyping, Product design adaptation) |
| Marketing Authorization (including Benefit-risk assessment, Benefits and risks weighing, Patient trade-off understanding, Subpopulation identification, Early access, Labelling optimization) |
| Post-marketing (including Product acceptance, Product innovation, Indication expansion, Risk assessment, Risk weighing) |
| Unknown |
